# The application value of high-frequency ultrasound in the feasibility assessment of endoscopic retrograde appendicitis therapy in children with appendicitis

**DOI:** 10.1038/s41598-023-46387-3

**Published:** 2023-11-07

**Authors:** Xiaoya Guo, Hengli Yang, Ping Zhao, Jinghua Li, Lingchao Zeng, Chunhui Wang, Yilin Yang, Ruijing Yang

**Affiliations:** 1 Department of Ultrasound Diagnosis, Tangdu Hospital of Air Force Military Medical University, Xi’an, China; 2 Department of Ultrasound Diagnosis, The Second Affiliated Hospital of Xi’an Medical College, Xi’an, China; 3 Department of Pediatrics, Tangdu Hospital of Air Force Military Medical University, Xi’an, China

**Keywords:** Diseases, Gastroenterology, Risk factors

## Abstract

Acute appendicitis is one of the common acute abdominal diseases in pediatrics. However, the implementation of radiological examination guided endoscopic retrograde appendicitis therapy (ERAT) in adults is limited in children. Our previous research explored the non-invasive guidance of high-frequency ultrasound (HFUS) for ERAT and achieved good therapeutic effects. This study mainly focuses on exploring the application value of HFUS in the feasibility assessment of ERAT in children with appendicitis. 163 children with appendicitis received ERAT guided by HFUS were analyzed retrospectively. According to the parameters evaluated by HFUS before and during ERAT, the results indicated that the distance between the appendix orifice and the ileocecal valve significantly affected the time required for the guidewire to enter the appendix cavity (P < 0.05). The diameter and the texture of the fecalith, the thickness of the intestinal wall of the appendiceal orifice all had significant effects on the successful removal of the fecalith (P < 0.05). The success rate, treatment time and final flushing effect of the guidewire to reach the blind end of the appendix were significantly affected by the tortuosity of the appendix and whether there was adhesion with surrounding tissues (P < 0.05). HFUS can accurately assess the feasibility of ERAT in children with appendicitis.

## Introduction

Acute appendicitis is a common surgical disease and one of the most common acute abdominal diseases in children^1^. In the early years, the main method for the treatment of acute appendicitis has evolved from open appendectomy to laparoscopic appendectomy^2^. In recent years, with the rapid development and wide application of endoscopic technology, endoscopic treatment of acute appendicitis in adults has been widely carried out^3^. However, the application of this technology requires radiological examination for intraoperative guidance and posttreatment evaluation, which limits its application to the treatment of acute appendicitis in children.

Ultrasound has the advantages of no radiation, real-time convenience, and ease of operability. In 2018, our hospital began to use HFUS combined with intraoperative intracavitary contrast-enhanced ultrasound imaging to accurately guide the ERAT in children with appendicitis. Most cases have achieved good results, however, the success of ERAT and the likelihood of efficacy are also affected by many factors in the local area of the appendix. The purpose of this study was to retrospectively analyze the children with appendicitis treated by ERAT in our hospital, to evaluate different parameters of the appendix before operation by HFUS, and to compare and analyze the impact on the difficulty of ERAT, so as to more accurately guide the clinical rational ERAT treatment of appendicitis in children.

## Results

After comprehensive analysis of the relevant data and treatment of the children in this study, we drew the following results:

### Comparison of the time for the guidewire entering the appendix cavity under different shapes of the appendix

**Figure 1:**
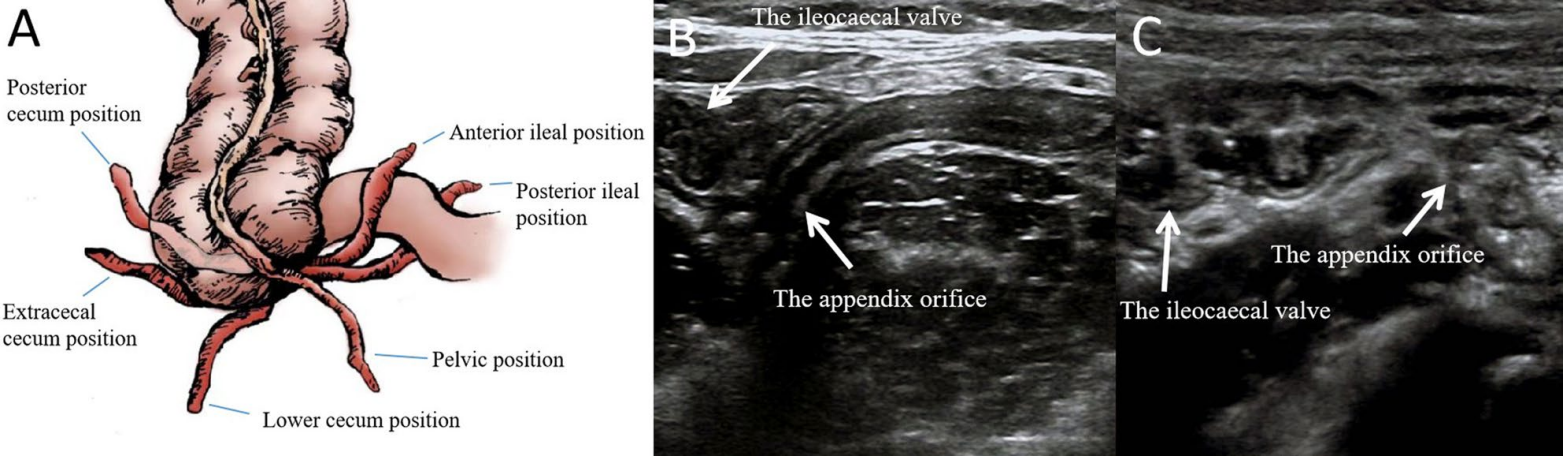
The diagram of different appendix positions and the different distance between the appendix orifice and the ileocaecal valve under HFUS. (**A**) The diagram of different appendix positions. (**B**) The distance between the appendix orifice and the ileocaecal valve was < 2.0 cm under HFUS. (**C**) The distance between the appendix orifice and the ileocaecal valve was ≥ 2.0 cm under HFUS.

The results showed that in 163 children, the position of the appendix was mainly pelvic position, followed by posterior ileal and lower cecum position, while anterior ileal, posterior cecum and extracecal cecum position were less (Table1, Fig. 1A). In this study, there was no significant difference in the time taken for the guidewire to enter the appendix cavity in all appendix positions (P > 0.05), indicating that the orientation of the appendix tip had no significant effect on the guidewire to enter the appendix cavity (Table1). However, when the distance from the appendix orifice to the ileocaecal valve was ≥ 2 cm, the time taken for the guidewire to reach the appendix cavity was shorter than when the distance was less than 2 cm [(11.31 ± 1.56) min vs. (14.28 ± 0.84) min, P < 0.05] (Table1, Fig. 1B,C).

**Table 1:** Comparison of the time required for the guidewire to enter the appendix cavity between different groups.

	The common appendix positions (the orientation of the appendix tip)						P	The distance between the appendix orifice and the ileocaecal valve		P
	Pelvic position (n = 98)	Posterior ileal position (n = 37)	Lower cecum position (n = 20)	Anterior ileal position (n = 6)	Posterior cecum position (n = 1)	Extracecal cecum position (n = 1)		< 2 cm (n = 21)	≥ 2 cm (n = 142)	
The time taken for the guidewire to enter the appendix cavity (min)	11.40 ± 1.53	11.32 ± 1.40	11.55 ± 0.60	11.33 ± 0.82	-	-	> 0.05	14.28 ± 0.84	11.31 ± 1.56*	< 0.05

* P < 0.05, with a statistically significant difference from the < 2 cm group.

### Comparison of successful removal of fecalith under different parameters

**Figure 2:**
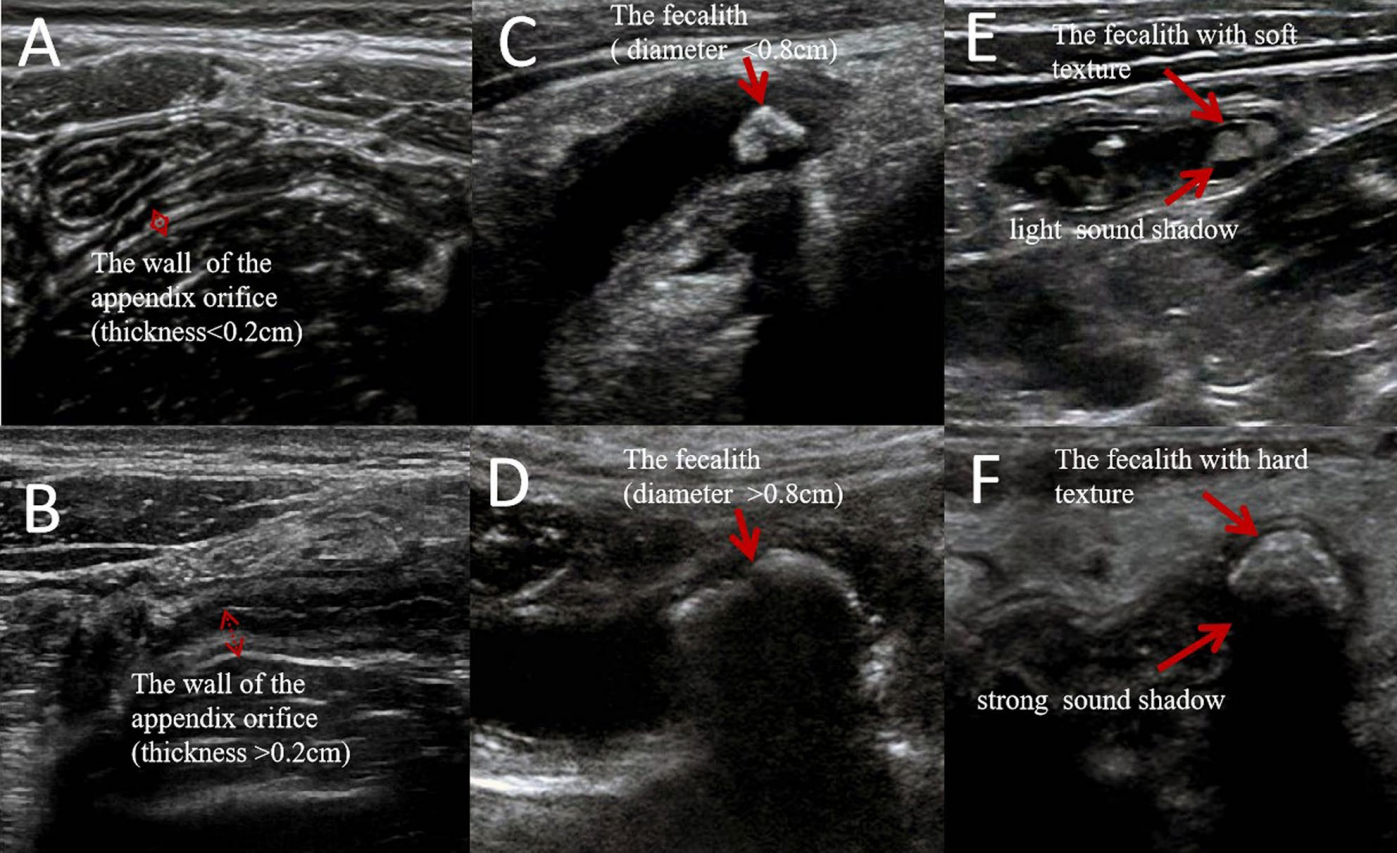
The different thickness of the intestinal wall of the appendix orifice and the different fecaliths in the appendix cavity under HFUS. (**A**,**B**) The thickness of the intestinal wall of the appendix orifice was < 0.20 cm and > 0.20 cm. (**C**,**D**) Fecaliths of different diameters (< 0.8 cm or ≥ 0.8 cm) in the appendix cavity. (**E**,**F**) The fecaliths with different textures and sound shadows in the appendix cavity.

**Figure 3:**
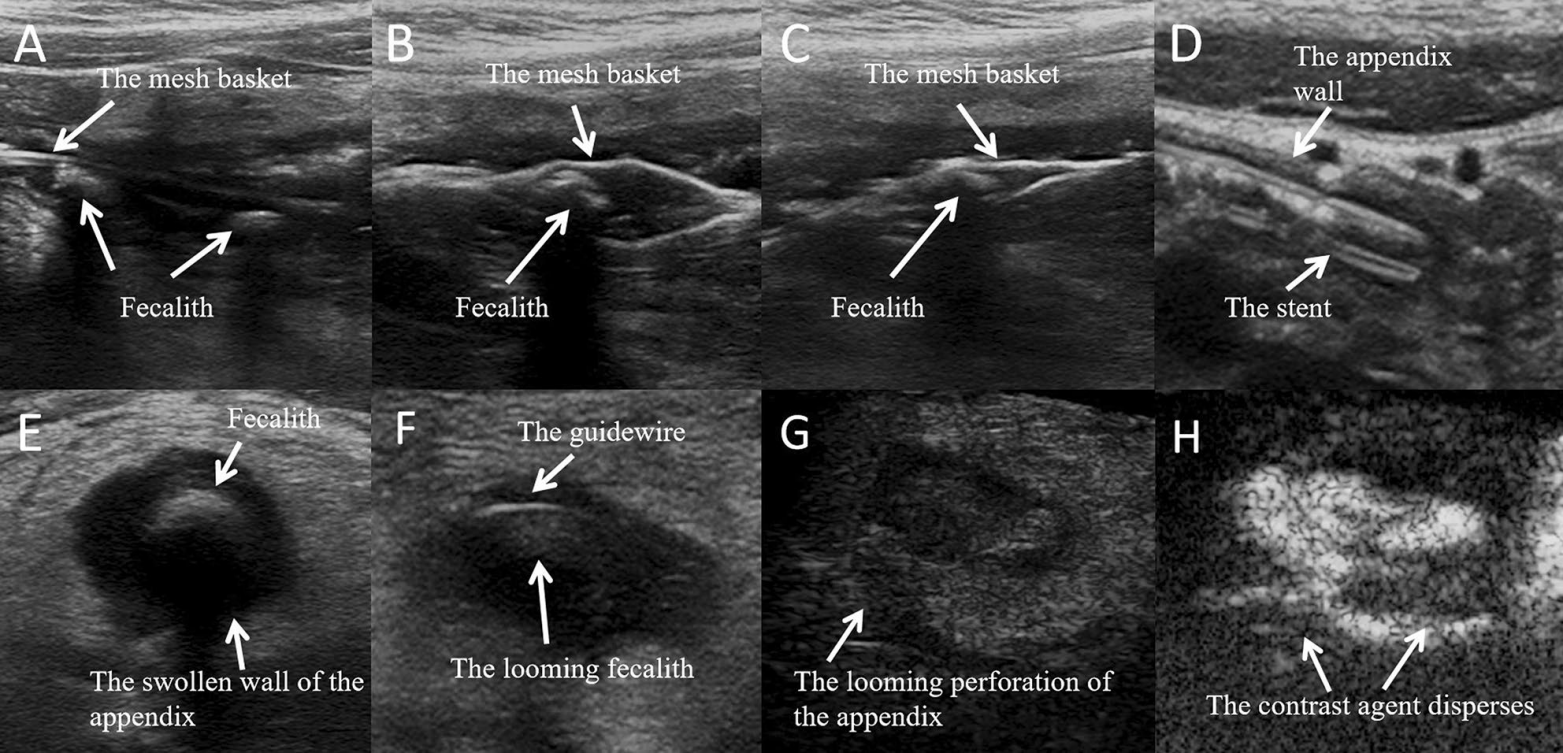
HFUS combined with intraoperative contrast-enhanced ultrasound imaging guided removal of fecalith. (**A**) The mesh basket slowly reached the fecalith (the diameter < 0.8 cm). (**B**) The mesh basket slowly opened. (**C**) The mesh basket rotated and tightened the fecalith. (**D**) The stent was placed after the fecalith was successfully removed. (**E**) There was a fecalith (the diameter ≥ 0.8 cm) with hard texture in the appendix cavity and the wall of appendix was significantly swollen. (**F**) The guidewire reached the fecalith. (**G**) Suspicious perforation of appendix wall during fecalith removal. (**H**) The intraoperative contrast-enhanced ultrasound imaging in the appendix cavity founded that the contrast agents dispersed around the appendix and determined that the appendix wall was perforated.

Of the 75 cases with fecalith in the appendix cavity, the fecalith was successfully removed in 49 cases, while removal failed in 26 cases. The results showed that the success of fecalith removal was significantly related to the thickness of the intestinal wall of the appendix orifice, the size of the fecalith, and the softness and hardness of the fecalith material. We compared the patients with a thickness of the intestinal wall of the appendix orifice < 0.20 cm vs. ≥ 0.20 cm and found that the former had a higher success rate of fecalith removal than the latter (74.47% vs. 50.00%, P < 0.05) (Table 2, Fig. 2A,B). Then, comparing patients with a fecalith diameter < 0.8 cm vs. ≥ 0.8 cm, we found the success rate of fecalith removal in the former was higher than that in the latter (80.70% vs. 16.67%, P < 0.05) (Table2, Fig. 2C,D, Fig. 3). When the diameter of the fecalith was ≥ 0.8 cm, HFUS showed that the appendix wall was significantly thickened and swollen, indicating that the operable space in the appendix cavity was correspondingly reduced. It was difficult to completely wrap or drag out the fecalith, regardless of whether the basket or balloon was used for fecalith removal. In this study, among the 26 children who failed to remove the fecalith, 5 patients had suspected perforation of the appendix wall due to HFUS during the operation, and further intraoperative contrast-enhanced ultrasound imaging in the appendix cavity confirmed that the contrast agents diffused around the appendix through the suspected defect of the appendix wall, indicating that the appendix wall was broken, and the children were immediately transferred to surgery for treatment. The prognosis of all children was good. The results determined that if the fecalith is forcibly removed, there will be a risk of perforation of the appendix wall (Fig. 3). Similarly, HFUS suggested that when the fecalith was soft, there was a light sound shadow behind it, and when the fecalith was hard, there was a strong sound shadow behind it. The results show that a lightly shadowed fecalith was associated with a higher success rate than a strongly shadowed fecalith (92.30% vs. 51.02%, P < 0.05) (Table2, Fig. 2E,F).

**Table 2:** Comparison of the success rate of fecalith removal under different parameters.

	The thickness of the intestinal wall of the appendix orifice (cm)		The diameter of the fecalith (cm)		The texture of the fecalith (sound shadow)	
	< 0.20 (n = 47)	≥ 0.20 (n = 28)	< 0.8 (n = 57)	≥ 0.8 (n = 18)	light sound shadow (n = 26)	strong sound shadow (n = 49)
The success number of fecalith removal (number of cases)	35	14	46	3	24	25
Successful rate (%)	74.47*	50.00	80.70^#^	16.67	92.30^^^	51.02

*P < 0.05, with a statistically significant difference from the ≥ 0.20 cm group.

^#^P < 0.05, with a statistically significant difference from the ≥ 0.8 cm group.

^^^P < 0.05, with a statistically significant difference from the strong sound shadow group.

### Comparison of treatment conditions for different degrees of tortuosity and adhesion with surrounding tissues

**Figure 4:**
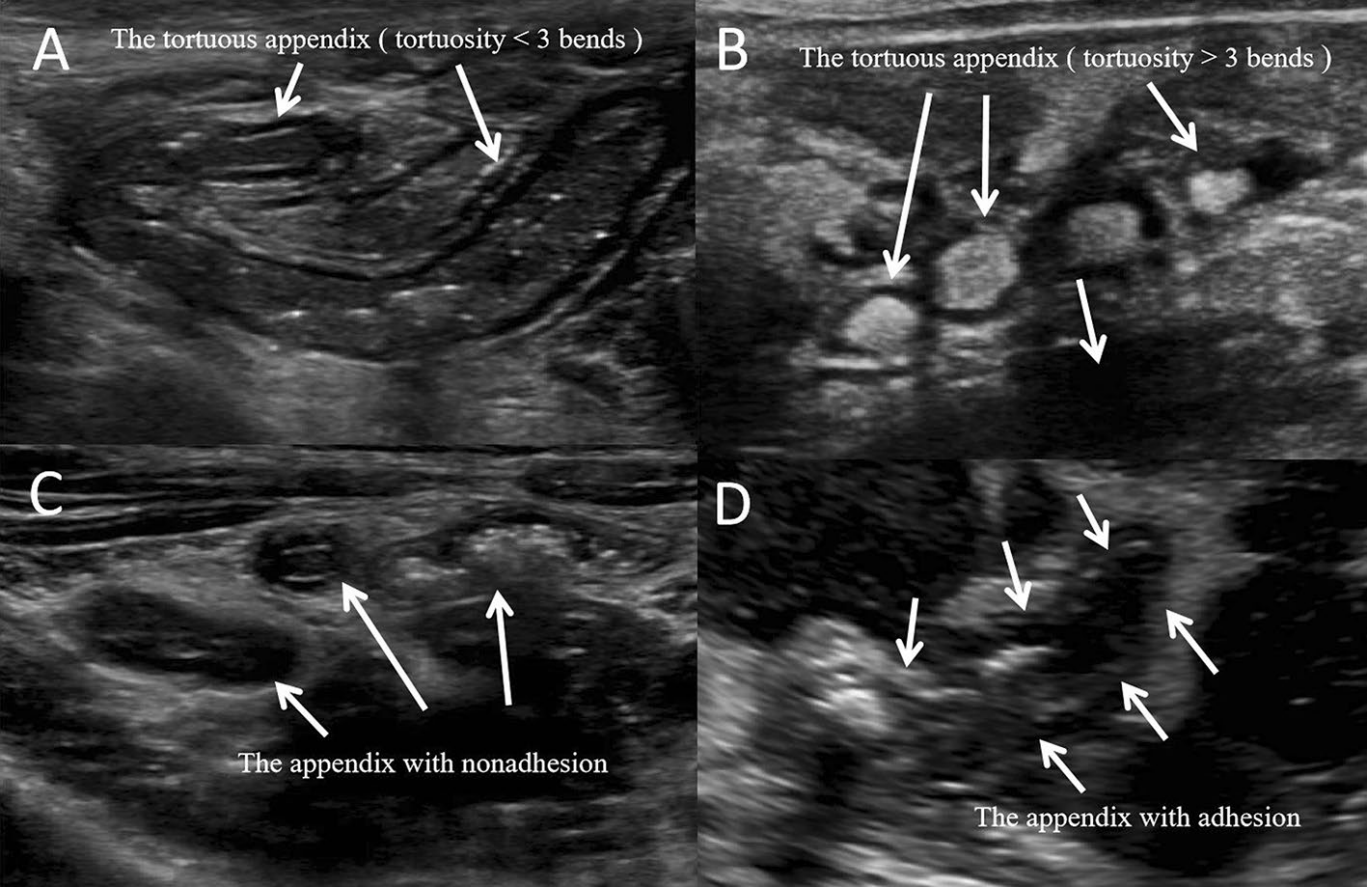
The appendix with different degree of tortuosity and adhesion. (**A**) The degree of tortuosity of the appendix was < 3 bends. (**B**) The degree of tortuosity of the appendix was ≥ 3 bends. (**C**) The appendix with nonadhesion, (**D**) The appendix with adhesion.

As shown in Table3, when the degree of the appendix tortuosity was < 3 bends, the success rate of the guidewire reaching the blind end of the appendix was higher than that with ≥ 3 bends (92.31% vs. 70.83%, P < 0.05) (Fig. 4A,B), and then, the time for the guidewire to reach the blind end of the appendix was shorter in the former than in the latter. ((6.95 ± 1.89) min vs. (8.98 ± 3.04) min, P < 0.05), but there was no significant difference in the total treatment time or the final flushing effect between the two groups (P > 0.05). The success rate of the guidewire reaching the blind end of the appendix in the nonadhesion group was significantly higher than that in the adhesion group (91.20% vs. 55.26%, P < 0.05) (Fig. 4C,D). The time it took the guidewire to reach the blind end of the appendix ((7.02 ± 1.34) min vs. (11.52 ± 4.04) min), the total treatment time ((47.77 ± 2.64) min vs. (56.09 ± 7.08) min), and the final flushing effect were also significantly different between the adhesion and nonadhesion groups (P < 0.05).

**Table 3:** Comparative analysis of the influence of different degrees of tortuosity and adhesion of the appendix on the treatment.

		The degree of tortuosity of the appendix		P	Appendix and surrounding tissue		P
		< 3 (n = 91)	≥ 3 (n = 72)		Nonadhesion (n = 125)	Adhesion (n = 38)	
The success rate of the guidewire reaching the blind end of the appendix (number of cases, rate)		84, 92.31%	51, 70.83%	< 0.05	114, 91.20%	21, 55.26%	< 0.05
The time to reach the blind end of the appendix (min)		6.95 ± 1.89	8.98 ± 3.04	< 0.05	7.02 ± 1.34	11.52 ± 4.04	< 0.05
The total treatment time (min)		48.47 ± 3.54	49.92 ± 6.13	< 0.05	47.77 ± 2.64	56.09 ± 7.08	< 0.05
The final flushing effect (number of cases, rate)	No residue	69,75.82%	51,70.83%	< 0.05	98,78.40%	5,13.16%	< 0.05
	Little residue	19,20.88%	13, 18.05%	< 0.05	11,8.80%	10,26.31%	< 0.05
	More residue	3,3.29%	8,11.11%	< 0.05	5,4.00%	23,60.53%	< 0.05

### Follow up

We conducted telephone or outpatient follow-up on 163 patients around 6 months after treatment. Among them, 16 patients were transferred to surgery, 23 patients experienced recurrent abdominal pain 1 to 3 months after leaving hospital and 17 patients were confirmed to have recurrent appendicitis by ultrasound examination, with a recurrence rate of about 11.5%. 6 patients had abdominal pain that lasted for half an hour to one hour, but spontaneously relieved after rest without relevant examination.

## Discussion

Acute appendicitis is one of the most common acute abdominal diseases^4^. In recent years, the incidence of pediatric appendicitis has been increasing. However, according to the literature, approximately 20% of acute appendicitis cases lack typical clinical features, and its clinical symptoms often do not match the actual condition, especially in elderly individuals, children, and pregnant women^5^. Appendicitis in children develops rapidly, from abdominal pain and appendicitis to purulence and perforation. The duration of the disease is only a few hours^6,7^. Although the appendix is small, it still plays an important role in the human body. The appendix has a certain endocrine function and immune effect in children, especially in adolescents^8,9^. Therefore, when appendix inflammation occurs in children, the desire to preserve the structure and function of the appendix is often stronger than that in adults^10^. It is particularly important to make an early and accurate diagnosis and then develop a rational treatment plan for acute appendicitis in children.

The advent of ERAT has opened a new chapter in the minimally invasive treatment of acute appendicitis^11^. Compared with the traditional X-ray guided ERAT, non-invasive ultrasound guided ERAT is one of the highlights of this treatment method in our hospital. Comprehensive analysis of the treatment of children with appendicitis who received ERAT in our hospital in the past 2 years showed that most of the treatment effects were significant. This article collected and analyzed the data of various parameters of appendicitis in the above children and evaluated its treatment effect, aiming to explore the value of HFUS in determining the feasibility of ERAT in children with appendicitis, summarize our experience, and provide a reference for clinical practice.

In most cases, the distance between the appendix orifice and the ileocaecal valve is (1.42 ± 0.62) cm, and it is theoretically believed that nearly 100% of people’s appendix orifices can be detected within 3.4 cm from the ileocaecal valve^12^. Therefore, in this study, 2 cm was set as the reference standard, the time for guidewire entering the appendix cavity was tracked, and the difficulty level was analyzed. Then we evaluated whether these numbers had any influence on the ERAT process. This results showed that when the distance between the appendix orifice and the ileocaecal valve was ≥ 2 cm, it was relatively easy for the guidewire to enter the appendix cavity. The reason for this is that when the distance between the two is less than 2 cm, the angle required for the guidewire to enter the appendiceal cavity while avoiding the ileocaecal valve is large, and the tip of the sphincterotomy knife is soft.

The appendix begins to appear at the top of the cecum approximately 8 weeks after the embryo begins developing. Due to the different growth rates of the two, the starting point of the appendix will move inward and rotate slightly, and the tip will be free, resulting in appendix position variations. Depending on the direction of the appendix tip, the common appendix positions are as follows: pelvic position, anterior ileal position, posterior ileal position, posterior cecum position, extracecal position, and intracecal position. Among them, the pelvic position is the most common, and it is extremely difficult to display the image of intracecal position^13–15^.In addition, due to the large anatomical variation of the appendix, there are also large differences in the position of the appendix^16^. The analysis of this study showed that the running position of the appendix, that is, the tip pointing direction, had little effect on the ERAT outcome, and there was no significant difference (P > 0.05). The reason for this result is that the base of the appendix is relatively fixed, the body and tip are free, and it is very mobile when there is no obvious adhesion to the surrounding tissue. During the process of the guidewire entering the appendix cavity, the tip direction changed with the guidewire direction or the shaking of the sphincterotomy knife.

In addition, there were 75 cases of fecalith formation in the appendix cavity in this study, and fecalith extraction failed in 26 cases, of which five cases had perforation during treatment. This results suggested that fecalith was often difficult to remove when it was large (≥ 0.8 cm in diameter) and hard in texture (with strong sound shadows behind). In appendicitis, the intestinal wall is swollen and brittle to varying degrees, the appendix cavity is narrowed, the power is relatively insufficient during normal saline flushing, and the large-diameter fecalith is difficult to move in the appendix cavity. When using the stone extraction mesh basket or balloon, which are often unable to be opened far or filling enough, resulting in failure of fecalith extraction. The soft-shaped fecalith can be slowly dissipated and crushed during the treatment process and can be discharged from the appendix orifice. For the fecalith with hard texture, the situation is the opposite of that of the fecalith with soft texture. Meanwhile, the degree of swelling of the intestinal wall of the appendix orifice also had a great influnce on the removal of the fecalith.

Finally, the degree of tortuosity and adhesion of the appendix affected the final flushing effect. Compared with appendiceal tortuosity ≥ 3 bends, appendiceal tortuosity < 3 bends made for a relatively simple operation, the success rate of the guidewire entering the blind end of the appendix was higher, and the time was shorter (P < 0.05). However, the base of the appendix is relatively fixed, the body and tip are free, and the mobility is greater when there is no obvious adhesion to the surrounding tissue. During the treatment process,the appendix can gradually straightened. Therefore, there was little difference between the two groups in the total treatment time or the final flushing effect(P > 0.05). If it adheres to the surrounding tissue to different degrees, the inflammation tends to be heavier, the mobility of the appendix is significantly reduced, the tissue is more brittle, and the difficulty and uncertainty of intubation are increased, and the technical requirements for the endoscopic operation are higher^17^ , Therefore, the indexes in the nonadhesion group were significantly different from those in the adhesion group in this study (P < 0.05).

So far, there are many reports on ERAT for appendicitis, but there are few reports on the feasibility assessment by ultrasound examination before treatment. This is also the innovation and highlight of this paper. However, there are still limitations to the study, such as the number of cases, which needs to be increased, and the grouping, which needs to be refined. With the continuous promotion and popularization of technology, as well as the accumulation and summary of experience, ERAT for pediatric appendicitis may have broad application prospects. Therefore, it is crucial to non-invasive evaluate the feasibility of ERAT for pediatric appendicitis before treatment. High frequency ultrasound can observe various indicators of appendicitis in children before treatment, and to some extent predict the treatment effect, providing a reference basis for clinical decision-making.

## Methods

### General information

The protocol of this research was approved by the institutional Review Board of Tangdu Hospital, Fourth Military Medical University Clinical Research Ethics Committee (the date of first registration: 18 /08/2018, the registration number: 201809–18). The trial was registered at Chinese Clinical Trials.gov: No. ChiCTR2000034562. Informed consent was obtained from each participant’s family according to the institutional guidelines and the clinical trial has been registered. This study used the CONSORT reporting guidelines, and cite them as: Schulz KF, Altman DG, Moher D, for the CONSORT Group. All methods involved in study were carried out according to institutional guideline.

This was a retrospective analysis of 163 children with appendicitis admitted to our hospital from January 2021 to April 2022. There were 78 males aged 3–15 years, with an average age of 5.63 ± 2.59 years, and 85 females aged 4–15 years, with an average age of 7.41 ± 3.35 years. The fecalith was found in the appendix cavity of 75 children, and there was no significant difference in sex or clinical data.

### Inclusion criteria

(1) Inclusion criteria: ① All patients had clinical symptoms of appendicitis, such as fever, abdominal pain, tenderness. ② The transverse section of the appendix showed round-like changes, with an outer diameter ≥ 0.6 cm or thickened appendix wall (≥ 0.2 cm). ③ fecalith, empyema, or fecal debris was observed in the appendix cavity. ④ The family members of the children refused surgical treatment and agreed to try ERAT.

(2) Exclusion criteria: ① Irregular hypoechoic or liquid dark areas around the appendix; ② fever or abdominal pain not caused by appendicitis; ③ contraindications for colonoscopy; ④ incomplete clinical data; ⑤ Abnormal liver, kidney or other functions.

### Ultrasonography before treatment

All children underwent HFUS examination before treatment, which was carried out by the same experienced sonographer. The Philips CX-50 ultrasound instrument linear-array probe (probe frequency: 12 MHz) was used for examination. The child was instructed to empty the bladder before the ultrasound examination and to lie in the supine position. Multiple sections were used for scanning. During the scanning process, the abdomen of the child was properly pressurized to reduce the interference of intestinal gas on the imaging. After obtaining a clear image, we measured the distance between the appendix orifice and the ileocaecal valve, the thickness of the intestinal wall of the appendix orifice, and observed the orientation of the appendix tip, the degree of tortuosity, and the adhesion with surrounding tissues. The diameter of every fecalith was measured, the texture was observed and detailed records were made.

### Treatment process

The ultrasonic instruments, probe conditions and imaging parameters used in the operation were the same as before. All children required intestinal preparation before treatment. 4 to 16 h before treatment, the child drank polyethylene glycol electrolyte solution (80 ml/kg) or performed 4 to 5 low-pressure cleaning enemas (250 to 500 ml of physiological saline for each enema). After intestinal preparation, dexmedetomidine nasal drops were administered, with a total dosage of 0.08–0.10 ml per 5 kg of body weight in both nasal cavities. Then, the child was placed in a left lying position, and when the child entered a sleep state, the endoscope was entered and the timing begined. The colonoscope was inserted through the cavity to the end of the cecum. The child was changed to the supine position to expose the appendix orifice. The guidewire and sphincterotomy knife slowly entered the appendix under ultrasound monitoring. At this time, we monitored by ultrasound the direction of the appendix, the degree of tortuosity and the position of the guidewire in real time. With a bow knife when appropriate, the appendix cavity was repeatedly flushed with normal saline while feeding the knife. Under the endoscope, a large amount of appendix cavity content could be seen being flushed out. When the knife for sphincterotomy reached the blind end of the appendix, flushing was continued until HFUS showed that there was no obvious fecal residue, fecalith, or other sonographic manifestations in the appendix cavity. When the washed-out normal saline became relatively clear, contrast-enhanced ultrasound in the appendix cavity was carried out for confirmation. For the fecalith that could not be flushed out, an endoscopic retrograde cholangiopancreatography stone extraction balloon or mesh basket was used to remove fecalith (Fig. 3). After confirming that the appendix cavity was rinsed clean, the sphincterotomy knife was withdrawn, and a plastic stent was placed along the guidewire under the guidance of HFUS monitoring. The endoscope was retracted from the body after ultrasound confirmation of proper stent position, and end the timing, which was the total duration of treatment. After 1–2 weeks of stent drainage, patients were followed up for ultrasound examination and blood routine examination. If the stent position and infection indicators were normal, the child was performed intestinal preparation and the stent was removed under colonoscopy. All patients were followed up for at least six months.

### Observed parameters during treatment

To observe the treatment process of ERAT guided by HFUS, different parameters and relevant data were recorded, including whether the guidewire successfully reached the blind end of the appendix, the time it took for the guidewire to reach the blind end of the appendix, the total treatment time, whether the fecalith was successfully removed, and the effect of appendix cavity flushing. The flushing effect of the appendix cavity was mainly divided into three situations according to the sonographic appearance, and detailed records were made: No residue: the liquid dark area in the appendix cavity was basically clear, no obvious lump-like echo was seen, and the blind end cavity of the appendix had no residue; little residue: a small accumulation of mass-like echo was seen in the cavity of the blind end of the appendix; more residue: a mass-like echo filling of the middle and distal appendiceal lumen was seen.

### Statistical analysis

All data is stored in the computer for subsequent statistical analysis.SPSS 25.0 was used for statistical analysis. Measurement data are reported as ${\overline{\text{x}}}$  ± s and were compared by Student’s t test. One-way analysis of variance was used to compare multiple groups. Count data are expressed as percentages and were compared by the χ^2^ test. P < 0.05 indicated a significant difference.

## Data Availability

The data that support the findings of this study are available from the corresponding author, upon reasonable request.
